# A Mutant of Uracil DNA Glycosylase That Distinguishes between Cytosine and 5-Methylcytosine

**DOI:** 10.1371/journal.pone.0095394

**Published:** 2014-04-16

**Authors:** Scott T. Kimber, Tom Brown, Keith R. Fox

**Affiliations:** 1 Centre for Biological Sciences, University of Southampton, Highfield, Southampton, United Kingdom; 2 Department of Chemistry, University of Oxford, Chemistry Research Laboratory, Oxford, United Kingdom; Louisiana State University and A & M College, United States of America

## Abstract

We demonstrate that a mutant of uracil DNA glycosylase (N123D:L191A) distinguishes between cytosine and methylcytosine. Uracil DNA glycosylase (UDG) efficiently removes uracil from DNA in a reaction in which the base is flipped into the enzyme’s active site. Uracil is selected over cytosine by a pattern of specific hydrogen bonds, and thymine is excluded by steric clash of its 5-methyl group with Y66. The N123D mutation generates an enzyme that excises cytosine. This N123D:L191A mutant excises C when it is mispaired with A or opposite an abasic site, but not when it is paired with G. In contrast no cleavage is observed with any substrates that contain 5-methylcytosine. This enzyme may offer a new approach for discriminating between cytosine and 5-methylcytosine.

## Introduction

Uracil, which is generated by deamination of cytosine [1] producing G.U mispairs, is removed from DNA by uracil-DNA glycosylase (UDG) [2]–[4]. UDG is highly specific for uracil and shows no activity towards any other base [5]; the base pair partner of the U is not recognised and the enzyme also acts on A.U base pairs that arise through misincorporation during DNA replication [6]. Uracil is flipped out of the duplex into the enzyme’s active site, followed by cleavage of the N-glycosidic bond [7]–[9]. This base flipping is aided by L191 that inserts into the DNA duplex [8], pushing out the uracil and increasing its lifetime in the active site [10]. The L191A mutant is less efficient at flipping out uracil [11], though the enzymatic activity can be rescued by pairing uracil with a bulky synthetic nucleoside that occupies the space of the base pair [10], [12]. Thymine is excluded from the active site of UDG by steric clash between its 5-methyl group and Y66 [9], [13].

**Figure 1:**
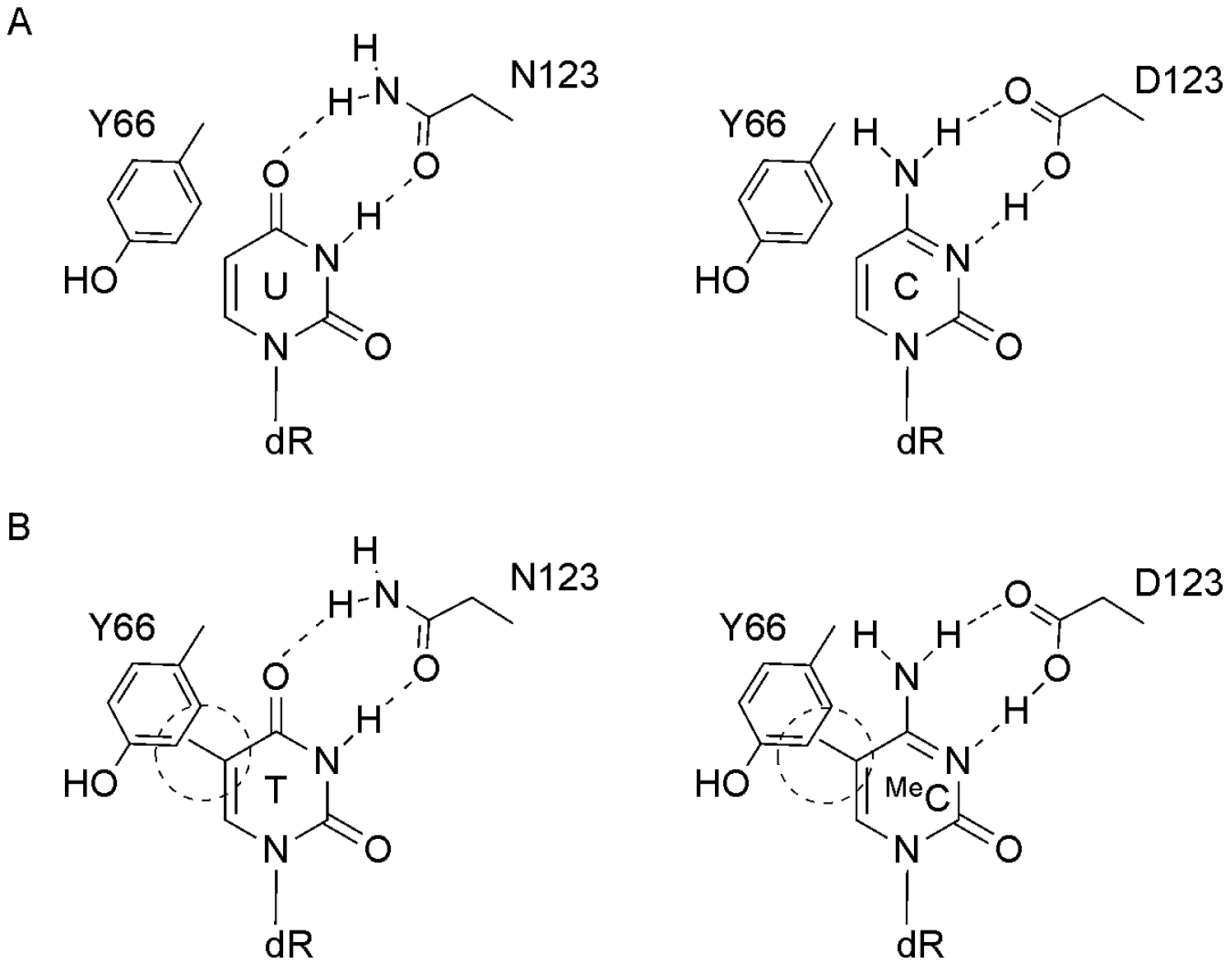
Interaction of UDG and CYDG with U, T, C and ^Me^C. A) Interaction of U with N123 in uracil DNA glycosylase and proposed recognition of C by D123 in the N123D mutant. B) Exclusion of T and ^Me^C caused by steric clash between their 5-methyl groups and Y66 (circled).

UDG’s remarkable specificity for uracil results from specific hydrogen bonding [14], [15] and shape complementarity [11], [12], [16], [17]. In particular N123 forms specific hydrogen bonds with O4 and N3 of uracil. Mutation of N123 to aspartate (N123D) alters the hydrogen bond donor-acceptor pattern, allowing for recognition of cytosine thereby generating a cytosine DNA glycosylase (CDG) as shown in Figure 1A [17]. The double mutant (N123D:L191A, designated as CYDG), is unable to excise cytosine from a G.C base pair [18]. It has been reported that this enzyme only shows CDG activity when C is paired with a bulky group such as pyrene, which forces the cytosine into an extrahelical conformation [10], [12], [18]. This mutant still cleaves uracil at which it is at least 1000-fold less active than UDG. We reasoned that CDG should be able to discriminate between cytosine and 5-methylcytosine (^Me^C) by the same mechanism that UDG discriminates between U and T (Figure 1B).

Cytosine methylation, especially at CpG sites, acts as an epigenetic marker which affects gene expression and regulation. The most commonly used methods for detecting 5-methylcytosine are direct sequencing after treatment with bisulphite [19] or protection from cleavage by methylation sensitive restriction enzymes [20], [21]. We have therefore explored whether CYDG can discriminate between C and ^Me^C, in the same way that UDG discriminates between U and T (Figure 1). We have determined the cleavage selectivity of CYDG and show that it can remove cytosine, but not methylcytosine, when it is mispaired or opposite an abasic site.

## Materials and Methods

### Preparation of Enzymes

The sequence of *Escherichia coli* UDG was cloned between the EcoRI and HindIII sites of pUC18. Site-directed mutagenesis generated the L191A mutation, which was followed by the N123D mutation. The sequence was then subcloned into pET28a and inserted between the NdeI and EcoRI sites. The enzyme (CYDG) was expressed in BL21(DE3)pLysS cells, which were induced with 0.2 mM IPTG for three hours. The cells were lysed by sonication, purified using a Ni-NTA (His Trap FF Crude; GE Healthcare) and eluted in 250 mM imidazole. The enzyme was concentrated and further purified using a 20 mL 10000 MW Vivaspin column (Fisher Scientific). This produced CYDG that was about 95% pure, as estimated by SDS polyacrylamide gel electrophoresis, with a yield of 1.5 mg per litre culture.

### Preparation of Oligonucleotides

Oligonucleotides were synthesized on an Applied Biosystems ABI 394 automated DNA/RNA synthesizer on the 0.2 or 1 µmol scale using standard methods. Phosphoramidite monomers and other reagents were purchased from Applied Biosystems or Link Technologies. The pyrrolidine anthraquinone phosphoramidite was purchased from Berry & Associates. Each 31 mer oligonucleotide was radiolabelled at its 5′-end with γ-^32^P[ATP] using T4 polynucleotide kinase (New England Biolabs), purified by denaturing PAGE, and resuspended in 10 mM MES pH 6.3 containing 25 mM NaCl and 2.5 mM MgCl_2_. These were mixed with an excess of the unlabelled complementary oligonucleotides and annealed by slowly cooling from 95°C to 4°C.

### Enzyme Cleavage

Radiolabelled DNA (approximately 50 nM) was incubated with CYDG (typically 1.25 µM) for up to 24 h, removing samples at various time intervals. The reaction was stopped using 10% piperidine (v/v) and heated at 95°C for 20 min to cleave the phosphodiester backbone. The samples were lyophilised, resuspended in 5 µL loading buffer (80% (v/v) formamide, 10 mM EDTA, 10 mM NaOH and 0.1% (w/v) bromophenol blue) and run on a 12.5% denaturing polyacrylamide gel containing 8 M urea. The gel was then fixed, dried, subjected to phosphorimaging and analysed using ImageQuantTL. Experiments were performed in triplicate; k_cat_ values were determined from plots of percentage cleaved against time, using SigmaPlot, by fitting each set of data to a single exponential rise to maximum. These were then averaged and the rate constants are reported with ± standard deviation. The rate of cleavage of some substrates was very low (less than 10% cleaved after 24 hours incubation). In these instances an estimate of the rate constant was obtained from the fraction cleaved at a given time, assuming a simple exponential process.

## Results

### Generation of CYDG (N123D, L191A)

Initial attempts to prepare the N123D mutant of *E. coli* UDG, which should have CDG activity, were unsuccessful, confirming that this enzyme is cytotoxic in *E. coli* [17], [18]. The L191A mutant was therefore first introduced into UDG (generating UYDG [7]), which was followed by the second N123D mutation to produce CYDG. The mutations were generated in pUC18 and then subcloned into pET28a followed by expression of the protein in *E. coli*.

### Excision Properties of CYDG

**Figure 2:**
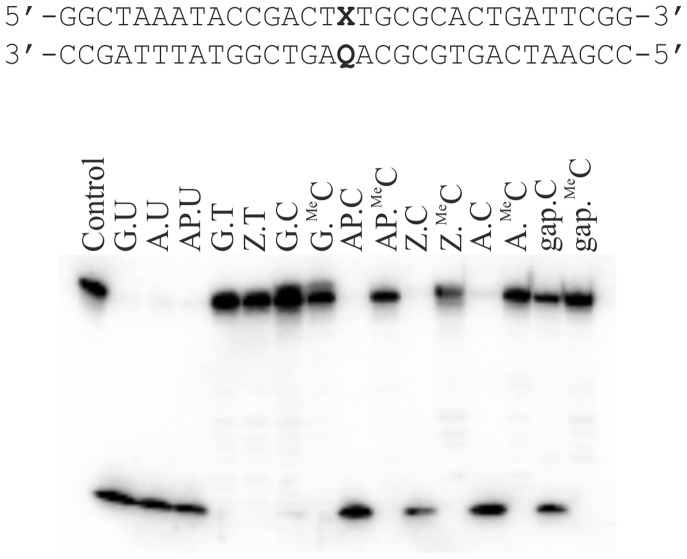
CYDG cleavage of 31 mer DNA fragments. The DNA fragments contained a central U, T, C or ^Me^C (X) opposite different bases (Q). The duplex substrates (∼50 nM), which had been labelled with ^32^P at the 5′-end of the upper strand, were incubated with ∼1.25 µM CYDG for 24 hours and then cleaved by heating at 95°C in 10% piperidine. The products were resolved on a 12.5% denaturing polyacrylamide gel.

The activity and specificity of CYDG were tested against a range of double and single stranded DNA templates. Synthetic 31 mer oligonucleotide substrates were designed so as to pair U, T, C or ^Me^C with G, A, AP (abasic site), Z (anthraquinone pyrrolidine) or a gap using two 15 mer oligonucleotides (Table 1). Previous studies have used a pyrene nucleoside [10], [12], [18] as a plug to force the base into the active site; we used anthraquinone pyrrolidine as a similar bulky nucleotide analogue. The results, after incubating all the substrates with an excess of the enzyme, are shown in Figure 2. Most importantly CYDG shows no activity against all the sequences that contain a central methylcytosine, confirming that the 5-methyl group of cytosine is excluded from the active site in a similar fashion to exclusion of the 5-methyl group of T by UDG. In contrast all the sequences with a central cytosine are cleaved, except when this is paired with guanine.

**Table 1:** DNA oligonucleotides used in this study to characterise the cleavage rates of CYDG.

Substrate	Sequence
G.U, A.U, A.C, AP.C, Z.C	5′-CCGAATCAGTGCGCA**X**AGTCGGTATTTAGCC3′-GGCTTAGTCACGCGT**Q**TCAGCCATAAATCGG
A.C(G)	5′-CCGAATCAGTGCGCG**C**GGTCGGTATTTAGCC 3′-GGCTTAGTCACGCGC**A**CCAGCCATAAATCGG
G.C	5′-CGAATAATTATATAA**C**ATATATATATTTAGC 3′-GCTTATTAATATATT**G**TATATATATAAATCG
gap.C	5′-CCGAATCAGTGCGCA**C**AGTCGGTATTTAGCC 3′-GGCTTAGTCACGCGT TCAGCCATAAATCGG
Long gap.C	5′-CCGTACTGAATCAGTGCGCA**C**AGTCGGTATTTACGATAGCC 3′-GGCATGACTTAGTCACGCGT TCAGCCATAAATGCTATCGG
ssC(polyA)	5′-AAAAAAAAAAAAAAA**C**AAAAAAAAAAAAAAA
ssC(GAT)	5′-GGATAAATAGGGAGT**C**TGAGAAGTGATTAGG

The target bases are shown in bold and underlined; where X = U or C and Q = G, A, AP (abasic site) or Z (anthraquinone pyrrolidine).

As expected, cleavage is observed when C is place opposite the bulky anthraquinone analogue, as previously observed with a pyrene nucleotide [18]. More surprisingly, cleavage is also observed when C is placed opposite an A, an abasic site or a gap, though there is no reaction with a G.C base pair. CYDG has residual activity against uracil, even when this is positioned opposite adenine, but showed no activity towards thymine in any base pair combination.

### Determination of k_cat_

**Figure 3:**
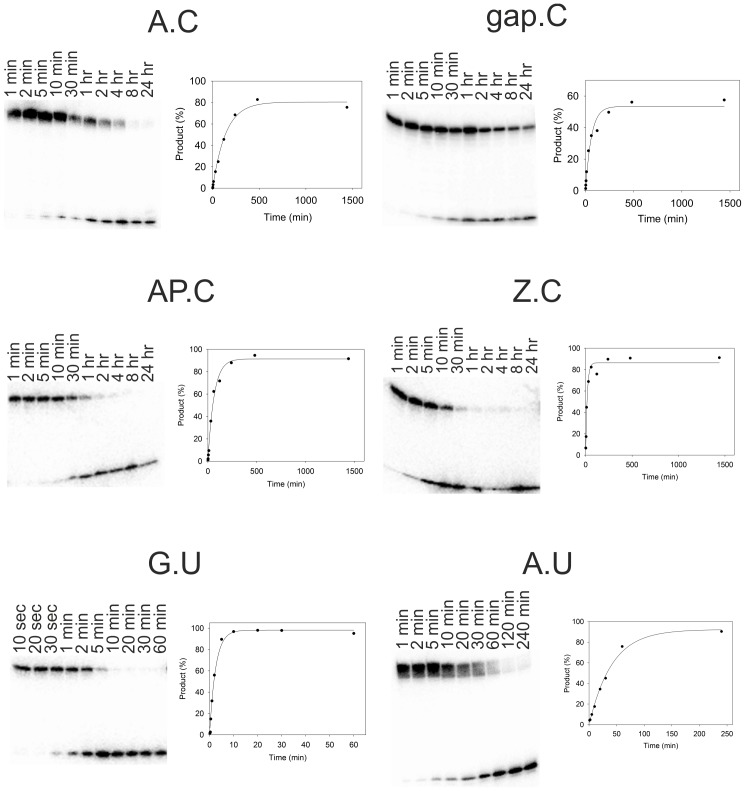
CYDG cleavage of fragments containing a central U or C opposite different bases. In each gel the ^32^P labelled duplex substrates (∼50 nM) were incubated with 1.25 µM CYDG for up to 24 hours and cleaved by boiling in 10% piperidine. The products were resolved on 12.5% denaturing polyacrylamide gels. The graphs are derived from phosphorimage analysis of the gels and show the rate of formation of the cleavage product. These are fitted with single exponential curves.

**Figure 4:**
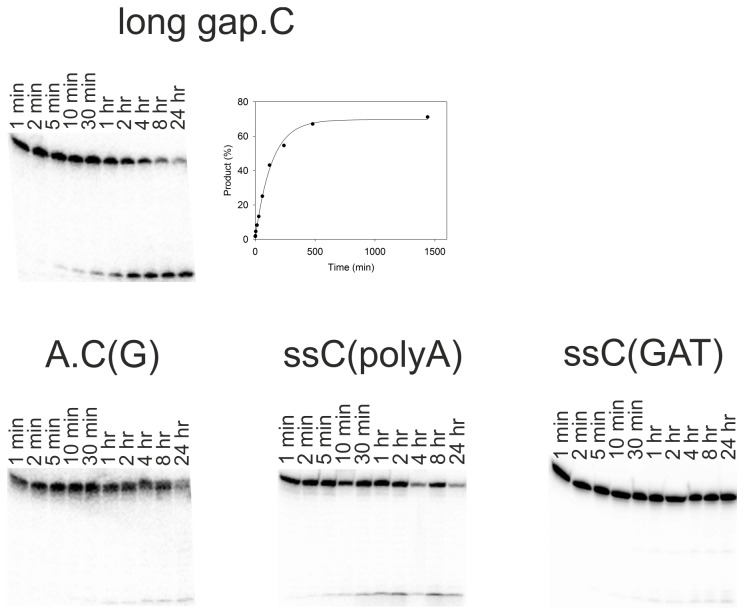
Kinetics of CYDG cleavage of fragments containing a central C. In each gel the ^32^P labelled duplex substrates (∼50 nM) were incubated with 1.25 µM CYDG for up to 24 hours and cleaved by boiling in 10% piperidine. The products were resolved on 12.5% denaturing polyacrylamide gels. The graph for long gap.C was derived from phosphorimage analysis of the gel and shows the rate of formation of the cleavage product; this is fitted with a single exponential curve.

In order to assess the best base pair combination for discriminating between C and ^Me^C we examined the kinetics of cleavage of C by CYDG when it is placed opposite various bases. Representative cleavage profiles are shown in Figures 3 and 4 and the data are summarised in Table 2. Reaction with the substrate containing a single A.C mismatch produced a single product at a rate of 0.006±0.001 min^−1^. The presence of a single product confirms that the enzyme does not cleave C when paired with G since this fragment contains several G.C base pairs. CYDG still cleaves at A.U and G.U, as previously reported [18], though this is much slower than native UDG at these sites. The excision of uracil from G.U (0.36±0.04 min^−1^) is approximately 60-fold faster, but the observation that cleavage at A.U (0.020±0.04 min^−1^) is about 20-fold slower than G.U suggests that the enzyme is best able to cleave C or U when they are in unstable (non-Watson-Crick) base pair combinations. Anthraquinone pyrrolidine was included opposite C so as to force the target base into an extrahelical conformation. This produced the fastest cleavage rate at C (0.10±0.02 min^−1^), faster even than A.U, though not as fast as at G.U; again no reaction is observed at Z.^Me^C. These results suggest that base pair stability plays a major role in determining the rate of cleavage. This is further confirmed by experiments with the sequence in which the A.C mismatch is flanked by G.C base pairs [A.C(G)] for which cleavage is reduced by about 100-fold compared to A.C flanked by A.T base pairs. Fast cleavage was also achieved with gap.C (0.016±0.002 min^−1^), which contains a gap opposite the C residue, allowing the unpaired cytosine to enter the active site of CYDG more easily. However, only 50% of this substrate was cleaved (Figure 3), while all other substrates were completely digested. This difference is probably due to the lower T_m_ of the duplexes formed with these split oligos, which is close to the reaction temperature. We therefore examined cleavage of an extended DNA substrate that contained an additional five base pairs on either side of the central C (long gap.C) (Figure 4). The extent of cleavage was improved to 80% with this longer substrate, though the reaction proceeded at a slightly slower rate. The lower cleavage efficiency may also be because CYDG binds with high affinity to the gap on the opposite strand, consistent with the observation that UDG has high affinity for AP sites protecting them from further mutagenesis during base excision repair [16].

**Table 2:** k_cat_ values for CYDG cleavage of the different DNA substrates.

Substrate	k_cat_ (min^−1^)	Rel
A.C	0.006±0.001	1.7
A.C(G)^1^	0.0001	∼0.02
AP.C	0.014±0.003	4.0
Z.C	0.10±0.02	29
G.C	ND	<0.001
gap.C^2^	0.016±0.002	4.6
Long gap.C	0.0072±0.0007	2.0
G.U	0.36±0.04	100
A.U	0.020±0.004	5.6
ssC(polyA)^1^	0.0003±0.0001	∼0.07
ssC(GAT)^1^	0.0001	∼0.02

The sequences of the oligonucleotides are shown in Table 1. No cleavage was observed for any substrate containing methylcytosine. ND - no cleavage detected after 24 hours. Values represent the average of three independent determinations ± standard deviations.

1 k_cat_ values were estimated from single time points at 24 hrs A.C(G), 60 mins ssC(polyA) and 4 hrs ssC(GAT).

2 Only 50% of the substrate was cleaved for gap.C. Rel indicates the cleavage rate relative to that of G.U (100).

We also examined the ability of CYDG to cleave Cs in single stranded DNA substrates (Figure 4). We used two substrates containing a single cytosine for these experiments; ssC(polyA) contains a single C residue within a polydA tract, while ssC(GAT) contains a single C within a mixed sequence of G, A and T. Although UDG cuts single-stranded Us faster than those paired with A or G [22], we observed only very slow cleavage of both single-stranded DNAs by CYDG.

## Discussion

These results show that CYDG, derived from *E. coli* UDG, is able to discriminate between cytosine and 5-methylcytosine; no activity against ^Me^C was detected in any of the substrates tested, while C is efficiently cleaved, except when it is paired with G. In UDG Y66 is positioned close to the 5 position of the pyrimidine base and the 5-methyl group is sterically excluded. Alteration of the hydrogen bonding pattern at N123 changes the base selectivity, but the mutant enzyme is still able to discriminate between pyrimidine and 5-methylpyrimidine. The lack of activity of CYDG against G.C base pairs therefore suggests the possibility of using this enzyme to probe the methylation status of a specific cytosine, by mispairing it with another base such as adenine.

CYDG cleaves cytosine when it is unpaired or mispaired, and the stability of the base pair determines the rate of cleavage [23], [24]. CYDG excised cytosine from Z.C faster than uracil from A.U, presumably because the mispaired cytosine is more easily forced into an extrahelical configuration than uracil in the Watson-Crick A.U pair. The faster cleavage of gap.C and AP.C occurs because there is no base opposite the C. If G.C base pairs flank the target cytosine then the rate of cleavage at A.C is dramatically reduced as a result of the increased local DNA stability [25] and the inability of CYDG to flip the base into the active site [10]–[12]. CYDG retains uracil DNA glycosylase activity despite the N123D mutation since free rotation of the aspartate side chain can still present the correct hydrogen bonding pattern for interacting with U [26]. Although the activity of CYDG is greatly reduced compared with wild type UDG, its catalytic activity is similar to that of many other DNA glycosylases [27]–[30].

The ability of CYDG to excise uracil from A.U but not cytosine from G.C suggests that this activity is dependent on the stability of the base pair and that the base can move into the enzyme’s active site when it is not involved in a stable base pair. The major role of L191 therefore seems to be to plug the space left after base flipping, rather than to actively assist the mechanism of base flipping itself [11]. The binding of CYDG to the duplex and the distortion it causes to the DNA [11], [16], [31] appears to be sufficient to destabilise an A.U but not G.C base pairs.

In summary we have shown that CYDG is able to discriminate between cytosine and 5-methylcytosine. Cytosine-DNA glycosylase activity is observed when C is unpaired or in an unstable (non Watson-Crick) base pair, while no activity is observed at ^Me^C in any base pair combination. This enzyme may offer an approach for discriminating between cytosine and 5-methylcytosine, in which the methylation status of a specific cytosine is probed by annealing it with an oligonucleotide that generates a mismatch, such as AC. A cytosine at this position will be cleaved while methylcytosine will not; PCR amplification of the reaction products can then be used to discriminate between the cleaved and uncleaved species.

## References

[1] Lindahl T, Nyberg B (1974) Heat-induced deamination of cytosine residues in deoxyribonucleic acid. Biochemistry 13: 3405–3410. 4601435 10.1021/bi00713a035

[2] Krokan HE, Bjoras M (2013) Base excision repair. Cold Spring Harb Perspect Biol 5: a012583. 23545420 10.1101/cshperspect.a012583 PMC3683898

[3] Zharkov DO, Mechetin GV, Nevinsky GA (2010) Uracil-DNA glycosylase: Structural, thermodynamic and kinetic aspects of lesion search and recognition. Mutat Res 685: 11–20. 19909758 10.1016/j.mrfmmm.2009.10.017 PMC3000906

[4] Friedman JI, Stivers JT (2010) Detection of damaged DNA bases by DNA glycosylase enzymes. Biochemistry 49: 4957–4967. 20469926 10.1021/bi100593a PMC2886154

[5] Lindahl T (1974) An N-glycosidase from Escherichia coli that releases free uracil from DNA containing deaminated cytosine residues. Proc Natl Acad Sci USA 71: 3649–3653. 4610583 10.1073/pnas.71.9.3649 PMC433833

[6] Tye BK, Nyman PO, Lehman IR, Hochhauser S, Weiss B (1977) Transient accumulation of Okazaki fragments as a result of uracil incorporation into nascent DNA. Proc Natl Acad Sci U S A 74: 154–157. 319455 10.1073/pnas.74.1.154 PMC393216

[7] Stivers JT, Pankiewicz KW, Watanabe KA (1999) Kinetic mechanism of damage site recognition and uracil flipping by Escherichia coli uracil DNA glycosylase. Biochemistry 38: 952–963. 9893991 10.1021/bi9818669

[8] Savva R, McAuley-Hecht K, Brown T, Pearl L (1995) The structural basis of specific base-excision repair by uracil-DNA glycosylase. Nature 373: 487–493. 7845459 10.1038/373487a0

[9] Mol CD, Arvai AS, Slupphaug G, Kavli B, Alseth I, et al (1995) Crystal structure and mutational analysis of human uracil-DNA glycosylase: structural basis for specificity and catalysis. Cell 80: 869–878. 7697717 10.1016/0092-8674(95)90290-2

[10] Jiang YL, Kwon K, Stivers JT (2001) Turning on uracil-DNA glycosylase using a pyrene nucleotide switch. Journal of Biological Chemistry 276: 42347–42354. 11551943 10.1074/jbc.M106594200

[11] Jiang YL, Stivers JT (2002) Mutational analysis of the base-flipping mechanism of uracil DNA glycosylase. Biochemistry 41: 11236–11247. 12220189 10.1021/bi026226r

[12] Jiang YL, Stivers JT, Song FH (2002) Base-flipping mutations of uracil DNA glycosylase: substrate rescue using a pyrene nucleotide wedge. Biochemistry 41: 11248–11254. 12220190 10.1021/bi026227j

[13] Handa P, Acharya N, Varshney U (2002) Effects of mutations at tyrosine 66 and asparagine 123 in the active site pocket of Escherichia coli uracil DNA glycosylase on uracil excision from synthetic DNA oligomers: evidence for the occurrence of long-range interactions between the enzyme and substrate. Nucleic Acids Research 30: 3086–3095. 12136091 10.1093/nar/gkf425 PMC135746

[14] Drohat AC, Stivers JT (2000) Escherichia coli uracil DNA glycosylase: NMR characterization of the short hydrogen bond from His187 to uracil O2. Biochemistry 39: 11865–11875. 11009598 10.1021/bi000922e

[15] Drohat AC, Xiao G, Tordova M, Jagadeesh J, Pankiewicz KW, et al (1999) Heteronuclear NMR and crystallographic studies of wild-type and H187Q Escherichia coli uracil DNA glycosylase: electrophilic catalysis of uracil expulsion by a neutral histidine 187. Biochemistry 38: 11876–11886. 10508390 10.1021/bi9910880

[16] Parikh SS, Mol CD, Slupphaug G, Bharati S, Krokan HE, et al (1998) Base excision repair initiation revealed by crystal structures and binding kinetics of human uracil-DNA glycosylase with DNA. EMBO J 17: 5214–5226. 9724657 10.1093/emboj/17.17.5214 PMC1170849

[17] Kavli B, Slupphaug G, Mol CD, Arvai AS, Peterson SB, et al (1996) Excision of cytosine and thymine from DNA by mutants of human uracil-DNA glycosylase. EMBO J 15: 3442–3447. 8670846 PMC451908

[18] Kwon K, Jiang YL, Stivers JT (2003) Rational engineering of a DNA glycosylase specific for an unnatural cytosine:pyrene base pair. Chem Biol 10: 351–359. 12725863 10.1016/s1074-5521(03)00077-2

[19] Shapiro R, Braverman B, Louis JB, Servis RE (1973) Nucleic acid reactivity and conformation. II. Reaction of cytosine and uracil with sodium bisulfite. J Biol Chem 248: 4060–4064. 4736082

[20] McClelland M, Nelson M, Raschke E (1994) Effect of site-specific modification on restriction endonucleases and DNA modification methyltransferases. Nucleic Acids Res 22: 3640–3659. 7937074 10.1093/nar/22.17.3640 PMC308336

[21] Cedar H, Solage A, Glaser G, Razin A (1979) Direct detection of methylated cytosine in DNA by use of the restriction enzyme MspI. Nucleic Acids Res 6: 2125–2132. 223125 10.1093/nar/6.6.2125 PMC327840

[22] Panayotou G, Brown T, Barlow T, Pearl LH, Savva R (1998) Direct measurement of the substrate preference of uracil-DNA glycosylase. J Biol Chem 273: 45–50. 9417045 10.1074/jbc.273.1.45

[23] Krosky DJ, Song F, Stivers JT (2005) The origins of high-affinity enzyme binding to an extrahelical DNA base. Biochemistry 44: 5949–5959. 15835884 10.1021/bi050084u

[24] Krosky DJ, Schwarz FP, Stivers JT (2004) Linear free energy correlations for enzymatic base flipping: how do damaged base pairs facilitate specific recognition? Biochemistry 43: 4188–4195. 15065862 10.1021/bi036303y

[25] Seibert E, Ross JB, Osman R (2002) Role of DNA flexibility in sequence-dependent activity of uracil DNA glycosylase. Biochemistry 41: 10976–10984. 12206669 10.1021/bi026121o

[26] Pearl LH (2000) Structure and function in the uracil-DNA glycosylase superfamily. Mutat Res 460: 165–181. 10946227 10.1016/s0921-8777(00)00025-2

[27] Roy R, Brooks C, Mitra S (1994) Purification and biochemical characterization of recombinant N-methylpurine-DNA glycosylase of the mouse. Biochemistry 33: 15131–15140. 7999773 10.1021/bi00254a024

[28] Neddermann P, Jiricny J (1994) Efficient removal of uracil from G.U mispairs by the mismatch-specific thymine DNA glycosylase from HeLa cells. Proc Natl Acad Sci U S A 91: 1642–1646. 8127859 10.1073/pnas.91.5.1642 PMC43219

[29] Bjelland S, Birkeland NK, Benneche T, Volden G, Seeberg E (1994) DNA glycosylase activities for thymine residues oxidized in the methyl group are functions of the AlkA enzyme in Escherichia coli. J Biol Chem 269: 30489–30495. 7982966

[30] Boiteux S, O’Connor TR, Lederer F, Gouyette A, Laval J (1990) Homogeneous Escherichia coli FPG protein. A DNA glycosylase which excises imidazole ring-opened purines and nicks DNA at apurinic/apyrimidinic sites. J Biol Chem 265: 3916–3922. 1689309

[31] Werner RM, Jiang YL, Gordley RG, Jagadeesh GJ, Ladner JE, et al (2000) Stressing-out DNA? The contribution of serine-phosphodiester interactions in catalysis by uracil DNA glycosylase. Biochemistry 39: 12585–12594. 11027138 10.1021/bi001532v

